# Improving Automatic Renal Segmentation in Clinically Normal and Abnormal Paediatric DCE-MRI via Contrast Maximisation and Convolutional Networks for Computing Markers of Kidney Function

**DOI:** 10.3390/s21237942

**Published:** 2021-11-28

**Authors:** Hykoush Asaturyan, Barbara Villarini, Karen Sarao, Jeanne S. Chow, Onur Afacan, Sila Kurugol

**Affiliations:** 1 School of Computer Science and Engineering, University of Westminster, London W1W 6UW, UK; B.Villarini@westminster.ac.uk; 2 Department of Radiology, Harvard Medical School and Boston Children’s Hospital, Boston, MA 02115, USA; Karen.Sarao@childrens.harvard.edu (K.S.); Jeanne.Chow@childrens.harvard.edu (J.S.C.); Onur.Afacan@childrens.harvard.edu (O.A.); Sila.Kurugol@childrens.harvard.edu (S.K.)

**Keywords:** cortex, DCE-MRI, GFR, kidney, medulla, MR urography, renal compartment, segmentation, time–intensity curve

## Abstract

There is a growing demand for fast, accurate computation of clinical markers to improve renal function and anatomy assessment with a single study. However, conventional techniques have limitations leading to overestimations of kidney function or failure to provide sufficient spatial resolution to target the disease location. In contrast, the computer-aided analysis of dynamic contrast-enhanced (DCE) magnetic resonance imaging (MRI) could generate significant markers, including the glomerular filtration rate (GFR) and time–intensity curves of the cortex and medulla for determining obstruction in the urinary tract. This paper presents a dual-stage fully modular framework for automatic renal compartment segmentation in 4D DCE-MRI volumes. (1) Memory-efficient 3D deep learning is integrated to localise each kidney by harnessing residual convolutional neural networks for improved convergence; segmentation is performed by efficiently learning spatial–temporal information coupled with boundary-preserving fully convolutional dense nets. (2) Renal contextual information is enhanced via non-linear transformation to segment the cortex and medulla. The proposed framework is evaluated on a paediatric dataset containing 60 4D DCE-MRI volumes exhibiting varying conditions affecting kidney function. Our technique outperforms a state-of-the-art approach based on a GrabCut and support vector machine classifier in mean dice similarity (DSC) by 3.8% and demonstrates higher statistical stability with lower standard deviation by 12.4% and 15.7% for cortex and medulla segmentation, respectively.

## 1. Introduction

Kidney-related disorders are reportedly a growing global problem, with approximately 2 million people dying from acute kidney injury and an estimated 5 to 10 million people dying annually from kidney disease [1]. Kidney damage leading to disease is caused by diabetes mellitus, hypertension and other chronic conditions. Furthermore, a recent study highlighted the detrimental impact of pain in patients with chronic kidney disease (CKD), where the occurrence of pain has been estimated at 50% to 70% in patients with advanced CKD [2].

Consequently, there is a growing demand for methods that accurately monitor and stratify renal function [3,4] and, thus, improve the assessment of disease prognosis, progression and treatment planning. Standard methods for the detection, diagnosis and stratification of decreased or abnormal renal function involve clinical chemistry measures. For example, a blood test provides an estimate of the glomerular filtration rate (GFR), which indicates the level of kidney function and determines the stage of kidney disease. One of the disadvantages of such clinical tests is the inability to target the disease location or provide a per-kidney assessment of GFR. Moreover, such conventional methods may overestimate GFR by around 10% to 20% [5].

In recent years, dynamic contrast-enhanced (DCE) magnetic resonance imaging (MRI) has gained credit in the medical community for accurate evaluation of localised renal function [6,7] without employing ionising radiation, from which measurements that pertain to morphology and function are computed. With the new advances of using non-Cartesian sampling methods for DCE-MR image acquisition, i.e., using a dynamic stack-of-stars radial sampling technique, high spatiotemporal resolution can be achieved for accurate function estimation. This new technique is simultaneously robust to respiratory motion. Recent results demonstrate that radial stack-of-stars acquisition enables accurate computation of kidney function markers from DCE-MRI [8,9,10].

In DCE-MRI scanning, the subject or patient receives an administration of gadolinium-based contrast agent into the bloodstream, which is then filtered through the kidney compartments. For every 3D image series acquired at different time points in DCE-MRI, the relative signal intensity change in the kidney over time reflects the rate of contrast transfer through the organ. Direct analysis of the DCE-MRI signal time curves from each kidney provides information that supports the diagnosis and classification of the disease severity. Furthermore, DCE-MRI scans that are processed through computer-aided systems using segmentation and a tracer kinetic model fitting can offer essential renal functional information, such as filtration rate. DCE-MRI is a unique modality that provides evaluation of both anatomy and function of kidneys at the same time using a single 6 min imaging sequence. The functional markers and anatomical evaluation are used together to make clinical decisions on whether the patient will need surgery.

The integration of MR urography (MRU) protocols involving DCE-MRI can add diagnostic value, allowing a thorough evaluation of renal function and anatomy with a single exam. This type of imaging, through the usage of a gadolinium contrast agent, is generally performed in the paediatric population, especially in the context of previously known congenital anomalies that have been identified prenatally or at birth. With the use of feed and wrap imaging, it is possible to perform MRU without sedation in babies [9]. Indeed, MRU can offer a detailed view of internal renal anatomy and function, which is a vital aid in evaluating a diverse range of congenital abnormalities of the kidneys and urinary tract. For instance, some children with prenatal hydronephrosis have non-obstructive dilatation of renal calyces that naturally lessens over time [11] without the need for any intervention. However, in other cases, there may be a ureteropelvic junction (UPJ) obstruction, which is a partial or total blockage at the junction of the kidney, pelvis and ureters. MRU can offer diagnostic guidance for obstruction that may lead to permanent kidney damage when there is a delay in intervention.

With the growing demand for DCE-MRI, the need to generate accurate clinical markers of kidney function from computer-aided systems is essential. A prerequisite includes segmenting the kidney parenchyma, which includes the cortex and medulla. One approach includes manual segmentation: an expert operator (e.g., a radiologist or radiographer) chooses 3D volumes in the DCE-MRI at specific temporal points when the contrast between individual, separate compartments in the kidney is highest, and then outlines a contour of each anatomical structure. However, manual segmentation is extremely time-consuming, fastidious and subject to high inter-observer and intra-observer variability. In contrast, an accurate and robust automated technique for segmenting the kidney parenchyma can potentially increase the usage of MRI for computing GFR and other clinical markers and can lessen the burden on radiologists and radiographers.

One challenge in accurate segmentation is the requirement of high temporal resolution, where each 3D volume is acquired in less than 3 s. This fast sampling reduces the quality of resultant DCE-MR images, limiting their spatial resolution and signal-to-noise ratio and resulting in images that include undersampling artefacts in the form of streaking. Moreover, despite the robustness to respiratory motion, the resultant images are blurred during heavy breathing, which is often observed in paediatric patients who exhibit nervousness during imaging. In addition, babies who are imaged during their sleep often move during the imaging, which results in signal dropout and artefacts.

Furthermore, regionally inhomogeneous intensity changes occur, particularly in the presence of diverse renal abnormalities during the passage of the contrast through the medulla and cortex.

Driven by a growing level of computer-aided systems in clinical practice, the research literature has seen a rise in automated methods for whole-kidney and renal compartment segmentation in the last decade. Commonly adapted approaches for whole-kidney segmentation in DCE-MR images include active contours [12] and spatial regularisation using discrete graph-cuts [13]. However, the disadvantages of such methods include limitations for diverse kidney structures and sizes with abnormalities. Moreover still, in contrast with methods for the whole kidney, the segmentation of renal compartments remains more challenging due to the high variation in size and structure and the irregularity in shape, especially with kidney abnormalities.

Combined with deep learning technology that utilises convolutional neural networks (CNNs), inspired by the work described in [14] and extending upon [15], this paper presents a fully modular framework with a translational impact for automatic segmentation of kidney parenchyma and renal compartments, i.e., the cortex and medulla, in DCE-MR images. The proposed approach addresses the challenges of segmenting imaged kidneys with poor function and compares with a recent state-of-the-art [16] that reports higher quantitative accuracies relative to other renal segmentation methods for DCE-MRI.

In the research literature for renal compartment segmentation, the authors of [17] proposed a semi-automated method for DCE-MRI data with a temporal dimension. In this approach, pixels relating to different internal kidney structures are classified in accordance with time–intensity curves using a *k*-means clustering algorithm. There exist limitations in automatically selecting the number of clusters in which spatially isolated greyscale pixels are misclassified. A self-supervised method reported in [18] automatically detects the initial seed points of internal kidney regions in the spatial domain and generates a supervised classifier using temporal information to segment the medulla and cortex. In some instances, the limitations of the weight function concerning the distance between the neighbouring greyscale pixels impact the classifier’s performance. The authors of [19] proposed a framework where the whole kidney is segmented using an approach based on the maximally stable temporal volume (MSTV) [20]. Afterwards, a *k*-means clustering is applied to separate segmented kidney voxels into multiple clusters that will define the separate renal compartments, including the cortex and medulla. In some cases involving clinically “abnormal” kidneys, the compartments are not always detected, and therefore, these intensity features do not fully satisfy the same feature rules applicable to clinically “normal” kidneys, which are relatively more consistent. Moreover, the authors suggested that the evaluated kidney imaging data lack a broad range of structural variability, which is essential in a clinical setting. The method proposed in [16] employs iterative graph cuts [21] and a random forest classifier to segment renal compartments in 4D DCE-MRI data, and it utilises a two-compartment model to estimate the GFR of each subject using both automatically and manually generated segmentation maps. In some instances, the automatic segmentation outcome fails to detect one kidney. Moreover, in a case where the kidney is detected, the lack of significant medulla enhancement and the clinically unusual location of the kidney result in a failed compartment segmentation outcome.

With the exception of the method described in [16], which utilised 26 paediatric scans, the performance of the methods described in [17,18,19] was evaluated on smaller datasets of 8, 10 and 14 predominantly clinically “normal” kidney cases, respectively. The scarcity of satisfactory renal compartment segmentation, particularly in paediatric cases from a broad age group with different abnormalities, drives the proposed fully modular and automated framework. Thus, the main contributions in this paper are as follows:

- The proposed framework will (a) address whole-kidney segmentation in clinically “normal” and “abnormal” DCE-MRI cases and (b) provide a strategy for renal compartment segmentation in cases involving (i) high temporal resolution and resultant undersampling artefacts and (ii) a diverse range of kidney abnormalities.
- The proposed framework is modular in design, such that each module can be used as an independent task to produce (a) whole-kidney segmentation and/or (b) renal compartment segmentation with a given reference of localisation (bounding box).
- The renal compartment segmentation technique improves rigorous discrimination between the medulla and cortex, particularly in “abnormal” paediatric cases compared to the state of the art, and it achieves a higher mean quantitative accuracy.
- To the best of our knowledge, this paper is one of the first studies to address renal compartment segmentation in a paediatric dataset of high variation in terms of age and kidney condition and to image the intra-spatial domain complexity due to varying artefacts. The proposed framework utilises a paediatric dataset acquired from patients aged from 2 months to 17 years, in which the anatomical shape of their kidneys ranges from clinically “normal” to sharp deformations of “abnormalities”.
- The improved segmentation of internal kidney regions could provide an opportunity to explore large-scale time–intensity curves of the medulla and cortex and, in doing so, could allow radiologists to differentiate clinically “normal” kidneys from conditions caused by obstruction of urine flow and dilation of the ureter.

The progression of this paper is structured as follows. Section 2 describes the proposed methods for whole-kidney and renal compartment segmentation in DCE-MRI 4D volumes. Section 3 analyses the quantitative accuracy results obtained by evaluating the segmentation outcomes against the expert-led, manual (delineated) ground truth. This section also highlights the computation of markers for clinical application following segmentation. Section 4 compares the segmentation quality in terms of quantitative accuracy and statistical robustness of the proposed methods against state-of-the-art methods in the research literature. Section 5 summarises the proposed method, including future work and the impact on clinical decision making.

## 2. Materials and Methods

Section 2.1 describes the dataset for training and testing purposes. The remainder of this section is divided into Section 2.2, which details the deep-learning-based approach for automatic segmentation in 4D DCE-MRI volumes, and Section 2.3, which explains the strategy for renal compartment segmentation by exploiting intensity-based contextual information in the temporal dimension.

### 2.1. Data

A dataset of 60 4D DCE-MRI scans of paediatric patients was employed, acquired at 3T (Tesla) for six minutes after injecting Gadavist (gadobutrol) using a motion-robust, radial stack-of-stars 3D FLASH sequence: TR of 3.56 milliseconds (ms), TE of 1.39 ms and Flip Angle (FA) of 12 degrees. A 4D volume is viewed as a stack of 3D volumes of the same region of interest captured over a period of time, and therefore, a 4D scan has a temporal dimension. Every 3D volume consists of 32 coronal slices of spatial size 224×224 (voxel size 1.25×1.25×3 mm). The ground-truth labels for whole and internal kidney regions were produced in the radiology department through expert-led manual delineation. The annotations were performed using the Insight Toolkit (ITK) by a highly skilled and experienced expert, and all annotations were verified by a senior radiologist.

The dataset included abdominal scans from a broad spectrum of ages (2 months to 17 years) with varying kidney conditions. Twenty-six DCE-MRI cases were evaluated for experimental purposes, ten of which were taken from patients who had received MRI scanning as part of their clinical protocol since 2017 and were diagnosed with conditions including hydronephrosis, multicystic dysplastic kidney (MCDK), obstruction of tubular dysfunction, and calyceal diverticulum. This particular group depicted kidney anatomy that deviated significantly from a clinically healthy shape. The remaining number of scans were taken from patients recruited under a protocol approved by an Institutional Review Board (IRB). This group of patients had already undergone contrast-enhanced MRI clinically, but they also received 6 additional minutes of research-based imaging of their kidneys within the same session to acquire the DCE-MRI. The acquisition protocol was optimised to achieve a mean temporal resolution of 3.3 s for the arterial phase (2 min) and 13 s for the remaining phase (4 min). The 4D dynamic image series were reconstructed offline using a compressed sensing algorithm to subsequently reduce streaking artefacts [22].

### 2.2. Automatic Kidney Segmentation

**Figure 1:**
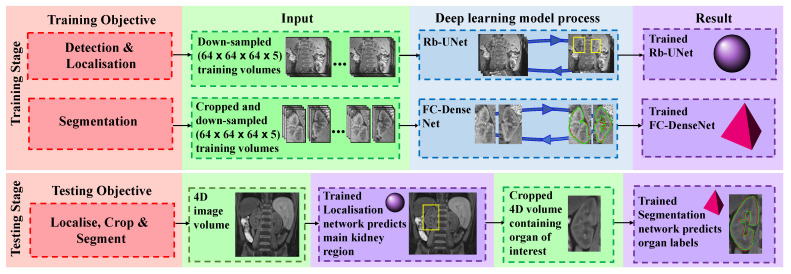
Overview of the proposed automatic kidney segmentation approach. The training stage simultaneously develops a network (3D Rb-UNet) for localising the organ and a segmentation network (3D FC-DenseNet) to predict the labels that correspond to kidney and non-kidney tissue. The testing stage processes an original scan (a 4D volume), performs a coarse segmentation to generate a bounding box capturing the main kidney region and then processes the cropped image volume to predict the labels of that organ.

An automatic kidney segmentation method embraces the advantages of 3D deep learning, in which volumetric contextual information is utilised. The proposed approach consists of a two-part process, where the first part detects and localises the kidneys, and the second part performs a detailed kidney segmentation [14]. The training stage and testing stage for each part are highlighted in Figure 1.

The first part of the training stage develops a model defined as 3D Rb-UNet. In this model, the residual connections introduced in [23], with the advantage of alleviating the vanishing gradient problem, are added at each block of a baseline U-Net [24] architecture, connecting the input of the convolutional layers at each scale to the outputs of the corresponding layer. Consequently, this bypass with identity connections for convolutional blocks at each scale improves the optimisation of convergence. Empirically tested, this model performed significantly better than other popular deep learning architectures [24,25,26] in localising each kidney, and is therefore employed at the testing stage when feeding an unseen (test) 4D DCE-MRI volume. The second part of the training stage develops a 3D fully convolutional dense net (FC-DenseNet) [27] using a uniformly cropped region of interest where the kidney is present, discarding background information that is unrelated to the organ. Here, the 3D FC-DenseNet provides a more diversified usage of concatenated features, which is appropriate for more refined, detailed voxel-wise segmentation.

In the testing stage, the fully trained 3D Rb-UNet performs a coarse kidney segmentation that represents the region of interest encapsulating the organ in an unseen DCE-MRI volume, and then generates a respective 3D minimum bounding box by fully enclosing this coarse segmentation against its “non-kidney” (background) class. Next, a cropped DCE-MRI volume, now containing only the kidney region of interest, is processed through the fully trained 3D FC-DenseNet model for voxel-wise predictions of intensity relating to the “kidney” (foreground) or “non-kidney” (background) class.

#### 2.2.1. Training Stage

The first phase of the training stage involves developing an object detection and localisation approach to eventually generate a bounding box over each kidney in the DCE-MRI volume and remove redundant information.

##### Detection and Localisation

The proposed network, 3D Rb-UNet, consists of an encoder of 4 blocks, a bottleneck and a decoder stage of 5 blocks extending upon the U-Net architecture. Each block in the encoder stage contains two 3×3×3 convolutions followed by a rectified linear unit (ReLu). The input of a convolutional layer is subsequently added to the output of the corresponding layer as a residual connection. Next, a 2×2×2 max-pooling is performed with strides of 2 for down-sampling and a dropout regularisation to counteract overfitting on high-resolution features. A bottleneck stage bridges the encoder and the decoder via two 3×3×3 convolutions, each followed by a ReLU and separated by a dropout regularisation. Next, each block in the decoder stage consists of a convolutional transpose of 2×2×2 by strides of 2 and two 3×3×3 convolutions, each followed by a ReLu and separated by dropout regularisation. Next, the layers with equal spatial resolution from the encoder stage are concatenated to their corresponding layers in the decoder stage to add high-resolution features to the latter stage. Next, the input of a convolutional layer is subsequently added to the output of the corresponding layer as a residual connection. Finally, batch normalisation enables faster convergence and reduced overfitting prior to a 1×1×1 convolution that reduces the number of output channels to the three classes in the last layer referring to the “background”, “right” and “left” kidney.

At the start of this training phase, the size of each image volume is reduced by downsampling to 64×64×64 to limit computational costs and still have sufficient resolution necessary for localisation and near-isotropic resolution across the *x*, *y* and *z* dimensions. Furthermore, experiments showed that reducing the temporal dimension from 150 samples along time (i.e., 150 image volumes in a 4D scan) to 5 samples, with the highest variance explaining 98% of the variance (i.e., 5 image volumes) using principal component analysis (PCA) [28], improved performance while avoiding memory overload. We first compute a list of the principal directions and magnitudes in each 4D volume and then transform the volume to align with these principal directions, reducing the temporal dimensionality. The spatial–temporal features are exploited during network optimisation despite the downsampling to identify unique features relating to kidney tissue. Moreover, data augmentation is generated as images scaled and translated in the range of [1, 4] pixels. Thus, the input data for the 3D Rb-UNet are 64×64×64 with 5 channels corresponding to the time dimension of the 5 volumes following the PCA. The weighted cross-entropy loss function, as in Equation (1), is employed to compensate for the class imbalance presented by diverse kidney structures and variability in the training data.

(1) $$W_{cross-entropy}=-\frac{1}{N}\sum_{i=1_{t}}^{N}w_{i}^{c}\left[\hat{p_{i}}\log p_{i}+\left(1-\hat{p_{i}}\right)\log\left(1-p_{i}\right)\right]$$

In Equation (1), *N* is number of voxels in an image volume, $p_{i}$ is the probability of voxel *i* belonging to the foreground in each output channel and $\hat{p_{i}}$ represents the true ground-truth label in the corresponding input channel. The $w_{i}^{c}$ is fixed as inversely proportional to the probability of voxel *i* belonging to the foreground class. Afterwards, softmax with weighted cross-entropy loss is used for the comparison of the network output and ground-truth labels.

##### Segmentation

Using the training data, every kidney is “cropped out” using the corresponding bounding boxes generated from the ground-truth labels. We initially interpolated and resampled each 4D volume to a standard temporal resolution and used 5 min as the maximum acquisition time. Hence, 50 samples from 5 min of acquisition were interpolated to ensure maximum variance of the time–intensity curves. Next, the dimensions of every image volume were reduced to 64×64×64 with a temporal dimension of 5 using PCA. The modified data was fed into the 3D FC-DenseNet to train the main segmentation model using the same weighted cross-entropy loss as in Equation (1).

The FC-DenseNet architecture builds upon DenseNet [25] to work as fully convolution networks (FCNs) by adding an upsampling path to compensate for the entire input resolution. The downsampling path performs a 3×3×3 convolution, followed by six dense blocks containing 4, 5, 7, 10, 12 and 15 layers. Each of the first five dense blocks follows a transition-down block consisting of batch normalisation, ReLU, 1×1×1 convolution, dropout and 2×2×2 max-pooling with a stride of 1. Thus, each layer in a dense block integrates batch normalisation, ReLU, a 3×3×3 convolution and a dropout regularisation. Next, the upsampling path consists of five transition-up blocks, each of which follows a dense block consisting of 12, 10, 7, 5, and 4 layers. Here, a transition-up block contains a 3×3×3 transposed convolution with a stride of 2. Finally, this path ends with a 1×1×1 convolution and a softmax function that predicts two classes of foreground (“kidney”) and background (“non-kidney”).

#### 2.2.2. Testing Stage

The fully trained 3D Rb-UNet performs a coarse segmentation, i.e., voxel-based prediction on the organ of interest using an unseen (test) 4D DCE-MRI volume. The reader is reminded that the prediction consists of two classes, “right” and “left”, relating to the default setting of two distinct kidneys, as well as the third class for “background”. Using the 4D test volume at its original size, two separate bounding boxes are generated to “crop out” the right and left kidneys. In cases where a missing kidney is identified, the corresponding bounding box is represented by the dimension of a single voxel.

The cropped test volume is fitted to the appropriate dimension of 64×64×64×5 and processed through the fully trained 3D FC-DenseNet, which performs detailed voxel-wise predictions of two classes, “kidney” (foreground) and “non-kidney” (background). Afterwards, each predicted organ binary mask is resampled to its original size and inserted into the corresponding spatial location in the test DCE-MRI volume.

### 2.3. Automatic Medulla and Cortex Segmentation

**Figure 2:**
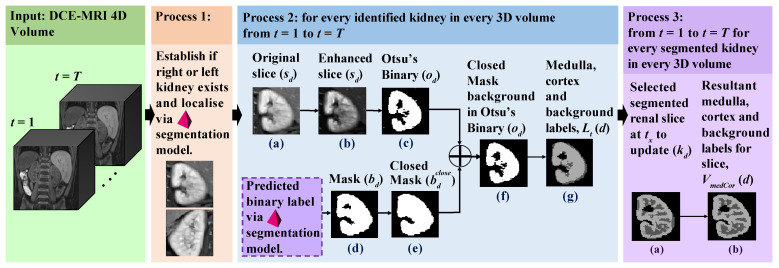
Overview of the proposed automatic renal segmentation approach. Using the input 4D DCE-MRI series, Process 1 detects the individual left and right kidneys (if present) via the automatic kidney segmentation approach. For each identified kidney, Process 2 performs medulla and cortex segmentation for all 3D volumes in the 4D DCE-MRI series. Process 3 generates the resulting single “optimum” volumetric medulla and cortex segmentation.

While the automatic kidney segmentation approach employs the latest advancements in deep learning architectures, the renal compartment segmentation approach exploits contrast-enhancing techniques to capture the internal kidney regions, including the medulla and cortex. The fully automatic approach proposed for renal segmentation consists of three main stages (processes), as summarised in Algorithm 1 and illustrated in Figure 2: Process 1 checks the existence of individual volumetric binary masks for the left and right kidneys and performs localisation and segmentation via the automatic deep-learning-based segmentation approach described in Section 2.2. Process 2 performs medulla and cortex segmentation for all 3D volumes in the 4D DCE-MRI volume *V*, where $V=\{V_{1},V_{2},\ldots ,V_{t},\ldots ,V_{T}\}$ and t∈Z:1≤t≤T. The resulting 4D volume, *L*, contains a sequence of 3D volumes, where $L=\{L_{1},L_{2},\ldots ,L_{t},\ldots ,L_{T}\}$, in which individual labels are assigned to the medulla, cortex and background. This stage serves as a prerequisite to identifying which volumes possess the highest-intensity contrast between the medulla and cortex for further processing in the next stage. Process 3 analyses every $L_{t}$ in *L* to identify the “optimum” medulla segmentation, and thus generates the resulting 3D volume segmentation, $V_{medCor}$, containing the renal compartment labels.
**Algorithm 1:** Medulla and Cortex Segmentation Process**Data:** DCE-MRI scan as a sequence of *T* 3D volumes:$V\;=\;\{V_{1},V_{2},\ldots ,V_{t},\ldots ,V_{T}\}$, where $V_{t}\in R^{H\times W\times D}$and *H* is the height, *W* is the width and *D* is the depth of each volume;Threshold parameters: δ (gain), μ (cut-off), γ (gamma correction);Range parameters: $r_{f}$, $r_{l}$;Whole-kidney segmented binary mask: $B\in Z_{2}^{H\times W\times D}$ where $Z_{2}=\left\{0,1\right\}$.**Result:**3D volume segmentation mask of the medulla and cortex in the whole kidney:$V_{medCor}\in Z_{3}^{H\times W\times D}$, where $Z_{3}=\left\{0,1,2\right\}$.**Process 1**: Establish if the *right* kidney exists and if *left* kidney exists.**Process 2**: Segment the medulla and cortex for all 3D volumes in *V* from t=1 to t=T.**Process 3**: Fuse the “optimum” medulla and cortex from all segmentationsover time, t=1 to t=T, into the final medulla and cortex 3D volume.

#### 2.3.1. Segmenting the Medulla and Cortex for All 3D Volumes in 4D DCE-MRI

After the detection of the left and right kidneys (if present), Process 2 segments the cortex and medulla in each 3D volume in the temporal series of volumes acquired. Process 2 begins by computing two distinct ranges that will contain a finite number of intensities for preliminary contrast enhancement at a later stage in the algorithm. The first range, $\left[1..p_{f}\right]$, is based on $p_{f}$, which represents the end position from the start of the slice depth, *D*, and where $p_{f}=r_{f}\times D$. The second range, $\left[p_{l}..D\right]$, is based on $p_{l}$, which represents the start position towards the end of *D* and where $p_{l}=r_{l}\times D$.

Next, given that $d\in \left[1..D\right]$, every original *d*-th 2D image slice, $s_{d}$, in every 3D volume in the 4D volume, *V* is analysed to eventually label each relevant pixel as the medulla or cortex. Figure 2, Process 2(a) shows an example of an original slice. From here, a number of markers to serve as “numerical guides” are computed: $a_{d}$, which contains the unique non-zero intensities in $s_{d}$, and the minimum and maximum values of $a_{d}$ as ${\left(a_{d}\right)}_{min}$ and ${\left(a_{d}\right)}_{max}$.

A number of contrast techniques that subsequently reduce noise artefacts are applied to manipulate the slice, $s_{d}$, through increasing contrast between the medulla and cortex, in which the former compartment’s intensities are, by default, darker (and numerically lower) than the latter compartment’s intensities.

Initially, for all intensities $s_{d_{i}}\in s_{d}$, where $i\in \left[1..H\times W\right]$ represents the intensity index position, the distribution in $s_{d}$ is updated using Equation (2):

(2) $$s_{d_{i}}\leftarrow s_{d_{i}}-{\left(a_{d}\right)}_{min}$$

under the condition that ${\left(a_{d}\right)}_{min}<s_{d_{i}}<{\left(a_{d}\right)}_{max}$ and $p_{f}\leq d\leq p_{l}$. Next, given that $d<p_{f}$ or $d>p_{l}$, a gamma correction is performed on the region of $s_{d}$ that contains non-zero pixel values, such that $s_{d}^{\gamma }\subset s_{d}$ and $s_{d}^{\gamma }\in R^{+}$ by using a non-linear transformation, as in Equation (3):

(3) $$G\left(s_{d}\right)={\left(\frac{s_{d}^{\gamma }}{255}\right)}^{\gamma }\times 255$$

where the value of gamma, $\frac{3}{2}\leq \gamma \leq \frac{7}{4}$, darkens the original brighter regions in $s_{d}$.

An application of contrast enhancement [29] amplifies the intensity variation to “enhance” the medulla regions in $s_{d}$ by applying a sigmoidal transformation, as in Equation (4):

(4) $$E\left(s_{d}\right)=\frac{1}{1+\exp\left[\delta (\mu -s_{d})\right]}$$

where δ is the gain, which controls the actual contrast, and μ is the cut-off value representing the normalised greyscale value about which the contrast level is changed. Figure 2, Process 2(b) shows an example of an enhanced $s_{d}$.

With $s_{d}$ having undergone a number of intensity-enhancing transformations, Otsu’s method [30] is applied to binarise $s_{d}$. Otsu’s method is an adaptive thresholding algorithm that finds the optimal threshold value in $s_{d}$, defined as $\sigma_{b}^{2}\left(\tau \right)$. From here, the binarised image of $s_{d}$ is defined as follows:

(5) $$o_{d}=s_{d}>\sigma_{b}^{2}\left(\tau \right)$$

in which pixels of value 0 represent the medulla and pixels of value 1 represent the cortex, as shown in Figure 2, Process 2(c). The following stages of Process 2 manipulate the labels in $o_{d}$ to generate the preliminary medulla and cortex labels:

1. The segmented binary mask of the kidney from Section 2.2 is defined as $B\in Z_{2}^{H\times W\times D}$, where $Z_{2}=\left\{0,1\right\}$, as shown in Figure 2, Process 2(d). Here, a 2D image, $b_{d}\subset B$, is fully closed to obtain $b_{d}^{close}$, as shown in Figure 2, Process 2(e).
2. Possible false positives in $o_{d}$ are eliminated by updating the background in $o_{d}$ to the same background as in $b_{d}^{close}$, as shown in Figure 2, Process 2(f).
3. If the initial pixel value is 0 and 1 in $o_{d}$ and $b_{d}$, respectively, then this pixel is labelled as “medulla”, as shown in dark grey in Figure 2, Process 2(g). Otherwise, this pixel is labelled as “cortex”.

Finally, $o_{d}$, now containing the updated medulla, cortex and background labels, is set to $L_{t}\left(d\right)$, where $L=\{L_{1},L_{2},\ldots ,L_{t},\ldots ,L_{T}\}$.

#### 2.3.2. Generating the “Optimum” Medulla and Cortex 3D Volume

After the completion of Process 2, the 4D volume of *L* now contains *T* medulla and cortex segmented 3D volumes. Process 3 aims to generate the “optimum” renal compartment 3D volume segmentation by analysing every slice, $l_{d}$, in $L_{t}$ over the temporal period, t=1 to t=T. First, the ranges $\left[1..p_{f}\right]$ and $\left[p_{l}..D\right]$ are established similarly to in Process 2. Next, as shown in Figure 2, Process 3(a), a 2D labeled image $k_{d}\subset L_{t_{x}}$ is selected, where $t_{x}=x\times T$ and where *x* is a constant. The labels in $k_{d}$ are updated by analysing against $l_{d}$. For every $l_{d}$, the following markers that serve as numerical “guides” are computed:

- Total area where $l_{d_{i}}>0$ as $area_{d}=\left|{\left\{l_{d_{i}}\right\}}_{i\in \{1,\ldots ,H\times W\}}\right|$.
- Medulla area in $l_{d_{i}}$ as $med_{d}=\left|{\left\{l_{d_{i}}\right\}}_{i\in \{1,\ldots ,H\times W\}}\right|$.
- Percentage of medulla in total kidney area, $r_{MA}=\frac{med_{d}}{area_{d}}\times 100$.

Next, for every $l_{d}\left(i\right)\in l_{d}|\left\{l_{d}\left(i\right)=“medulla”\;\wedge \;k_{d}\left(i\right)=“cortex”\right\}$, the label in $k_{d}$ is updated to “medulla” given a set of satisfied conditions, such that $\left(\alpha <r_{MA}<\beta \right)$, where α and β are constants and $\left(p_{f}\leq d\leq p_{l}\right)$. Figure 2, Process 3 (b) shows an example of an updated $k_{d}$.

The cortex labels in $k_{d}$ are improved by considering the closed binarised 2D image at $t_{x}$ as $\Theta_{t_{x}}\left(d\right)\subset \Theta_{t}$ and the closed binarised 2D segmented image that was achieved using the automatic kidney segmentation approach, Y(d)⊂Y.

In order to boost the classification of accurate cortex labels, $Y^{out}\left(d\right)$ describes the difference between the dilation and erosion of Y(d). The value in $k_{d}\left(i\right)\in k_{d}$ is updated to “cortex”, under the condition that the following criteria are satisfied: Y(d,i)=“cortex”, $\Theta_{t_{x}}\left(d,i\right)=“background”$ and $k_{d}\left(i\right)=“medulla”$. Furthermore, the cortex labels in $k_{d}$ are updated to have the same cortex labels as in $Y{\left(d\right)}^{out}$.

Finally, the resultant $k_{d}$, now containing the updated labels for the medulla, cortex and background, is set to $V_{medCor}\left(d\right)$, where $V_{medCor}$ is the final medulla and cortex segmentation 3D volume.

## 3. Results

The segmentation framework was implemented using Python 3.0 and Matlab (Release 2016b) on a PC running on an NVIDIA Quadro P6000 GPU via Centos 7.0 OS. The implementation is available at https://github.com/med-seg/kidney-mc Accessed on: 27 November 2021.

### 3.1. Experimental Setup

In order to develop the 3D FC-DenseNet deep learning model, as in Section 2.2, the training and testing dataset were split into 34 and 26 image volumes, respectively. The training dataset combined both clinically “normal” and “abnormal” cases. The optimisation algorithm used for training was Adam [31] with an initial learning rate of 0.0001. The hyperparameters included reduction rate (0.8), growth rate (12), momentum (0.9), weight decay $\left(10^{-8}\right)$ and dropout rate (0.2); the learning rate drop period was 50 and the learning rate drop factor was 0.5. The maximum number of epochs was 400 and the size of the mini-batch to use for each training iteration was set to 4. The training time for the network was approximately 3 h, and the testing time for a single DCE-MRI case was approximately one minute.

The thresholding parameters implemented in Process 2 were as follows: δ (gain) and μ (cut-off) values were 2 and 1.5, respectively. The value of γ (gamma correction) was 1.5. Empirically tested, the parameters for ranges $r_{f}$ and $r_{l}$ were 0.3 and 0.7, respectively. The parameters for ranges $r_{f}$ and $r_{l}$ implemented in Process 3 were 0.3 and 0.7, respectively. The medulla-to-full-area percentages of α and β were 30 and 60, respectively. Furthermore, the value of *x* in selecting $t_{x}$ lay between 0.25 and 0.50.

#### Evaluation

The performance of the proposed approach was evaluated using the dice similarity coefficient (DSC), precision (PC) and recall (RC). Should *G* represent the volumetric ground truth and should *S* represent the corresponding automatic segmentation labels, the DSC accuracy of *S* relative to *G* is defined as: $DSC\;=\;2\left(\left|G\cap S\right|\right)/\left(\left|G\right|\;+\;\left|S\right|\right)$. The precision normalises the true segmentation against the entire segmentation: $PC\;=\;\left(\left|S\cap G\right|\right)/\left|S\right|$. The recall (i.e., sensitivity) normalises S∩G against the ground truth, *G* and is defined as: $RC\;=\;\left(\left|S\cap G\right|\right)/\left|G\right|$.

### 3.2. Renal Segmentation

The proposed fully automated kidney segmentation method delivered a mean DSC ± standard deviation (SD) of 88.20 ± 6.41% for all 26 test cases. The relatively low standard deviation demonstrates statistical stability in the segmentation method, especially considering the diversity of kidney abnormalities and imaging artefacts. Considering a subset of 16 clinically “normal” cases achieved 89.77 ± 4.79% and evaluating a subset of 10 “abnormal” cases achieved 85.70 ± 7.75%. Table 1 highlights corresponding precision and recall accuracy scores, demonstrating the robustness in predicting true-positive labels and avoiding false-negative predictions.

**Figure 3:**
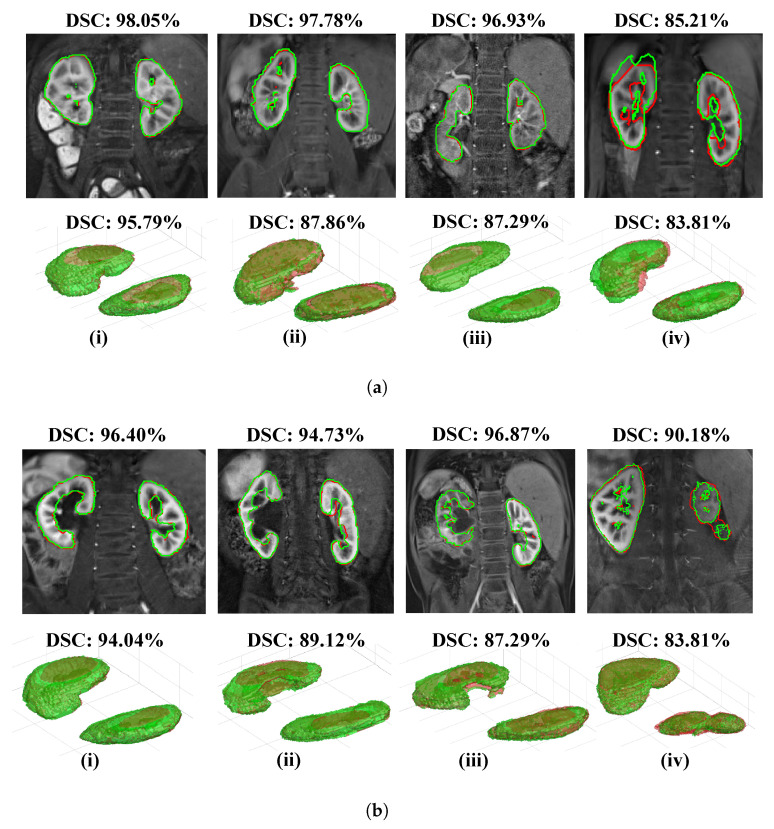
Whole-kidney segmentation results in eight different DCE-MRI scans (4D volumes). Every column corresponds to one MRI volume. The first row displays a sample DCE-MRI coronal slice with the segmentation outcome (green) overlapping the ground truth (red) and dice similarity coefficient (DSC). The second row displays a 3D reconstruction of the kidney and DSC. (**a**) Segmentations in four clinically “normal” cases; (**b**) Segmentations in four clinically “abnormal” cases.

Figure 3a and Figure 3b, respectively, show four different 3D segmentation reconstructions of “normal” and “abnormal” whole-kidneys (green) overlapping the ground truth (red), with an accompanying coronal slice highlighting the boundary contouring. As shown across the slices in the first row of Figure 3a, the change in greyscale intensity, noise and blurring impacted the rate of true-positive predictions, but continued to minimise false-negative predictions successfully. The slices in the first row of Figure 3b capture the true-positive predictions in a diverse range of abnormal kidney sizes and structures.

Considering all 26 test cases, the proposed fully automated renal segmentation method delivered a mean DSC ± SD of 72.34 ± 6.09% for all averaged medulla and cortex accuracy scores and revealed statistical significance using a permutation paired-sample test (p < 0.0001). Table 2 lists the respective individual DSC scores, reflecting significant robustness in the methodology considering the variety of kidney sizes and structures and imaging intensities, artefacts and textures.

**Figure 4:**
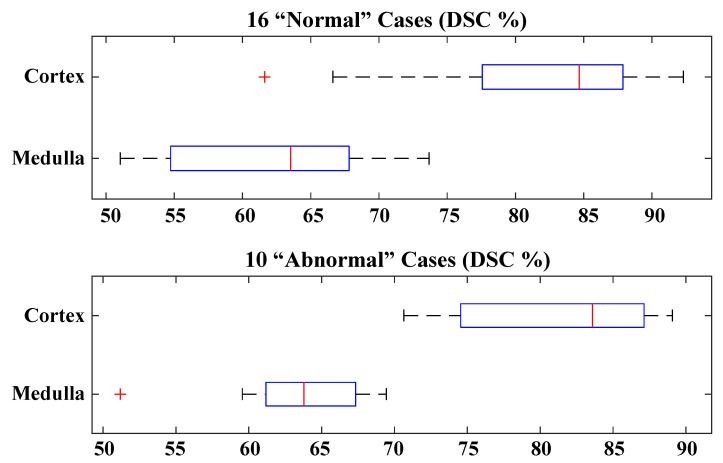
Box plots for two datasets depicting the medulla and cortex dice score coefficients (DSCs) for clinically “normal” and “abnormal” kidneys.

As shown in Table 3, evaluating a subset of 16 clinically “normal” cases achieved 62.40 ± 6.69% and 81.87 ± 8.21% for the medulla and cortex, respectively, and evaluating a subset of 10 “abnormal” cases achieved 63.41 ± 5.16% and 81.90 ± 6.31%. A box-and-whisker plot representation for both datasets is displayed in Figure 4. Analysing the “abnormal” cortex segmentation, a broader range between the median and lower quartiles in comparison to the “normal” segmentation confirms the higher degree of variation in the kidneys’ outer shapes and sizes. In contrast, a smaller interquartile range in the “normal” cortex segmentation reflects a lower degree of error between individual cortex segmentation cases. Due to the varied levels of motion-based and noise artefacts in the “normal” DCE-MRI dataset, there was a higher variation in corresponding medulla segmentation accuracies in comparison to the “abnormal” medulla accuracies.

**Figure 5:**
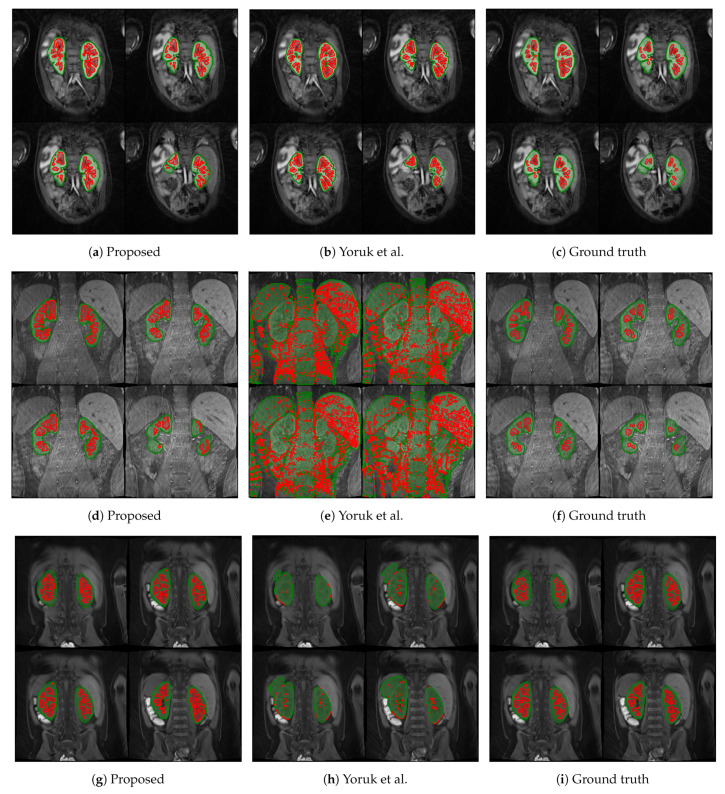
Medulla and cortex segmentation results of three different clinically “normal” kidneys. The first column (**a**,**d**,**g**) shows the results from the proposed approach; the second column (**b**,**e**,**h**) shows the respective results using the baseline approach from Yoruk et al. [16]; the third column (**c**,**f**,**i**) shows the respective ground truth.

**Figure 6:**
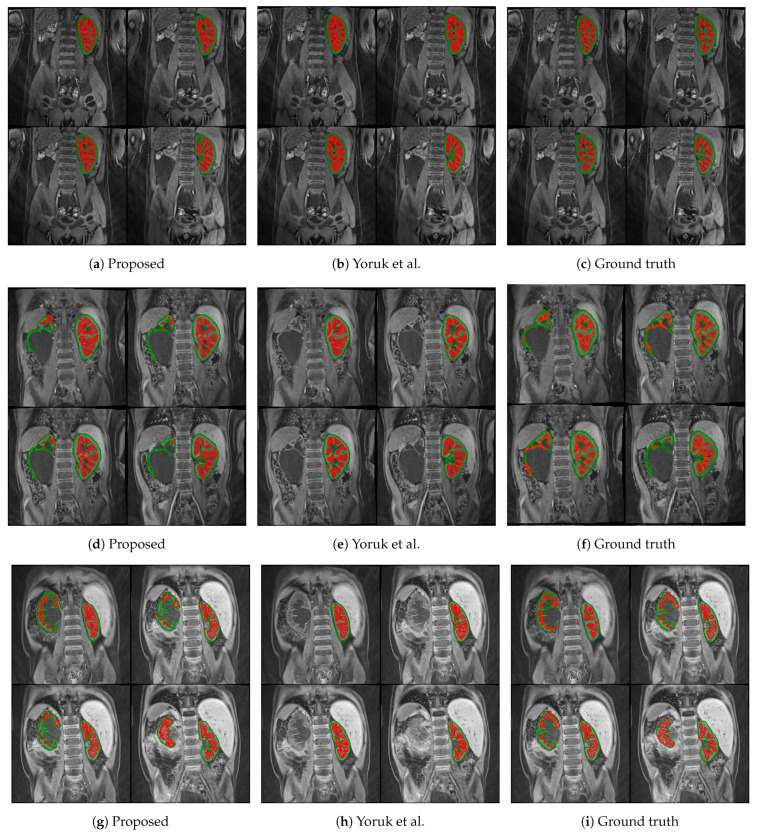
Medulla and cortex segmentation results of three different clinically “abnormal” kidneys. The first column (**a**,**d**,**g**) shows the results from the proposed approach; the second column (**b**,**e**,**h**) shows the respective results using the baseline approach from Yoruk et al. [16]; the third column (**c**,**f**,**i**) shows the respective ground truth.

The first columns of Figure 5 and Figure 6 display the medulla (red) and cortex (green) segmentation results in four coronal slices from a single DCE-MRI scan depicting clinically “normal” and “abnormal” kidneys, respectively. Using contrast enhancement via gamma correction and sigmoidal transformation maintained the contextual information while differentiating kidney regions; this occurred mainly in temporal instances where the contrast between DCE-MR imaged renal compartments was low but enough to capture boundary differences where the medulla and cortex edges were in contact.

#### Time–Intensity and Tracer Concentration Curves

**Figure 7:**
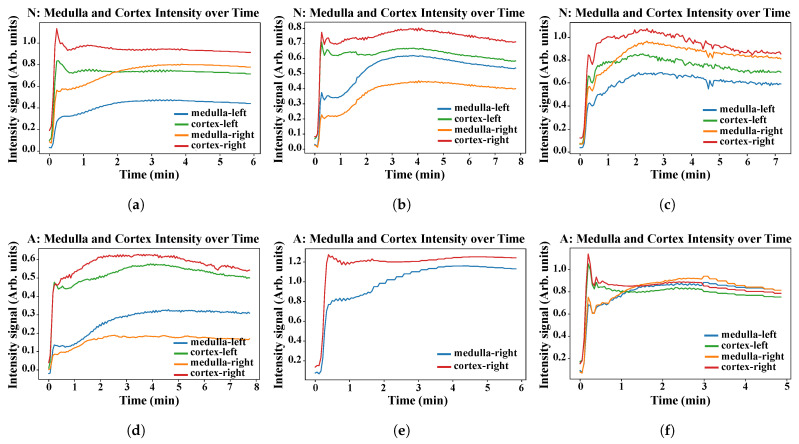
Graphs (**a**–**f**) represent clinically “normal” and “abnormal” cases (scans or 4D volumes), respectively. The relative contrast intensity enhancement of the (automatically segmented) medulla and cortex in both the left and right kidneys is shown over time (minutes).

**Figure 8:**
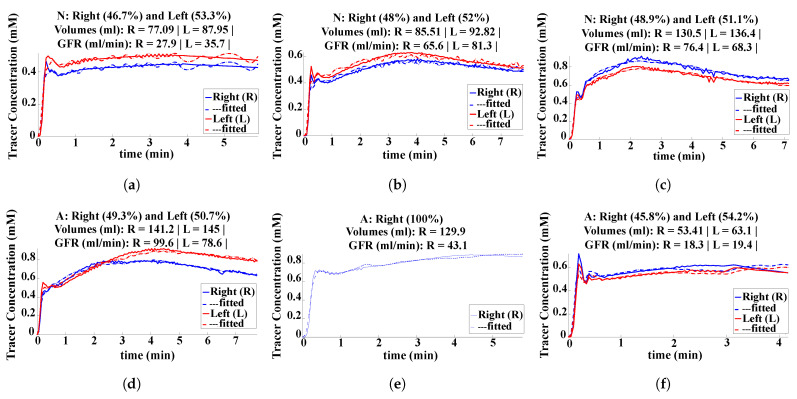
Graphs (**a**–**f**) represent clinically “normal” and “abnormal” cases (scans or 4D volumes), respectively.The tracer concentration is shown over time for both the right (blue) and left (red) kidneys; the corresponding kidney percentage, volume (mL) and GFR (mL/min) were computed to discern and evaluate separate kidney functions.

Figure 7 shows, in total, six examples of the relative contrast-enhancement time–intensity plots of clinically “normal” (a,b,c) and “abnormal” (d,e,f) medulla and cortex segmentation. Moreover, Figure 8 highlights six corresponding examples of the level of radioactive (tracer) concentration in kidney tissue over time, specifically in the clinically “normal” and “abnormal” segmentations. The segmented whole kidney can be used to estimate MRI-derived perfusion parameters and the GFR.

## 4. Discussion

In order to evaluate the proposed automatic segmentation model against the state of the art, the 3D U-Net serves as a baseline method to compare the proposed approach’s effectiveness and statistical stability. The encoder–decoder architecture in the 3D U-Net has served as the foundation for subsequent deep learning technologies [25,32,33] and was therefore chosen as a suitable comparative model.

Using 3D Rb-UNet, the localisation stage performs a coarse segmentation in order to identify the main region encapsulating the kidney and discard background information. Furthermore, we aim to limit computational costs and improve time efficiency, since the network’s primary input is 4D data.

The integrated 3D FC-DenseNet utilises the benefits of DenseNet and therefore has fewer parameters than 3D U-Net, and it avoids overfitting. Not only does the 3D FC-DenseNet extend upon DenseNet by adding an upsampling path to recover the full input spatial resolution, but this architecture also employs dense skip connections on skip pathways to improve gradient flow. Consequently, the temporal dimension of primary input data is harnessed, allowing deep feature supervision for learning kidney boundaries.

The identity shortcuts of the residual blocks in 3D Rb-UNet allow faster training and improved convergence in comparison with the 3D FC-DenseNet, which is excellent for localisation. In contrast, the 3D FC-DenseNet provides the advantage of higher capacity with multi-layer feature concatenation and achieves very detailed and fine boundary-preserving segmentation given localised kidneys as the primary input.

As shown in Table 1, the proposed model outperforms the baseline with prior localisation using 3D Rb-UNet by approximately 3.8% in mean DSC and demonstrates higher statistical stability by approximately 1.5%. Similarly, the proposed model surpasses the baseline approach by approximately 4.5% and 2.7% in mean DSC when evaluating the clinically “normal” and “abnormal” cases as two separate datasets, respectively. The robust consistency of the proposed approach is highlighted in a standard deviation that is relatively lower by approximately 3.7% and 1.2% in the “normal” and “abnormal” datasets, respectively.

The second and last column in Figure 3b highlights a relatively higher false-negative prediction of the right kidney, arguably proving a need to optimise data augmentation and training data where abnormalities are present in the imaged organ of interest. In addition, downsampling the input volume in the training stage of the segmentation model could easily result in the loss of contextual information and, thus, impact the network optimisation. It could be useful to incorporate a higher frequency of feature selection to reduce the high bias during network training while maintaining the computational costs that arise from downsampling.

The proposed renal segmentation approach outperforms a baseline method [16] for extracting the medulla and cortex by approximately 9.3% and 17.1% in mean DSC, respectively, and it boasts higher statistical stability by approximately 15.7% and 12.4%, as shown in Table 3. An available implementation [34] is utilised to reproduce the baseline method. Whereas the baseline method utilises computer vision to extract the whole kidney, the approach proposed in this report employs advancing deep learning to predict highly diverse kidney features, especially of abnormalities. As shown in Figure 6e,h, the algorithm in [16] fails to detect one of the clinically “abnormal” kidneys, whereas the proposed approach has accurately identified the entire organ, as illustrated in Figure 6d and Figure 6g, respectively. Moreover, the baseline approach completely breaks down in a clinically “normal” case, as shown in Figure 5e. In contrast, the robustness of the deep-learning-based model captures both kidneys before renal segmentation is performed, as shown in Figure 5d. A limitation of this particular case (Figure 5d) includes a relatively higher false-negative cortex prediction, resulting in a higher false-positive medulla compared to the ground truth in Figure 5f. Therefore, thresholding parameters would require optimisation to ensure a more robust generalisability. Thus, it would be helpful to expand upon nature-inspired algorithms such as the firefly and swarm intelligence algorithms to determine multilevel thresholds and enhance the compartment segmentation efficiency. In Figure 5h, the binary dilation and erosion strategy in the baseline approach predicts false-positive labels of renal parenchyma, whereas the proposed trained deep learning model accurately localises the kidney. Furthermore, the relatively high concentration of false-negative medulla labels demonstrates the limitations of incorporating the GrabCut and SVM classifier, as in [16]. In comparison, the proposed renal segmentation method exploits intensity enhancement throughout the full temporal dimension of the DCE-MRI to generate the “optimum” medulla labels for the resultant segmentation, as highlighted in Figure 5g.

### Application

The accurate segmentation of the whole and internal kidney regions can be used to extract clinically important markers of renal function. For example, these markers could optimise clinical decision making for whether a patient with hydronephrosis needs immediate surgery to preserve renal function, or whether a conservative method of treatment will be selected. This section provides two examples of clinical application in which the delineated kidney and renal compartments are used to generate graphical representations of kidney activity.

For example, as shown in Figure 7, the change in greyscale intensity over time indicates the rate at which the gadolinium contrast agent reaches the kidney and, thus, an insight into the condition of this organ. Moreover, it might be useful to explore a large-scale analysis of the medulla and cortex time–intensity curves against corresponding DCE-MRI scans in order to develop a method that guides the clinical classification of each kidney directly from the curves [35,36] and, thus, help to establish the likelihood of ureteral obstruction quickly. The respective comparison with ground-truth plots can be located in Appendix A.

Furthermore, the tracer concentration of the contrast in each kidney, which is dependent on properties of kidney tissue including perfusion, basement membrane and the cumulative concentration in arterial blood, is computed using the GRE sequence Bloch equations. As shown in Figure 8, the GFR is computed by fitting a kinetic tracer model to the signal averaged over each kidney, as described in [37]. For example, the kidney volume is used as a biomarker in autosomal dominant polycystic kidney disease (ADPKD), and GFR measurements are used to evaluate disease progression [38].

## 5. Conclusions

Kidney-based disorders are a growing global problem. Therefore, there is mounting demand for methods that accurately monitor, stratify and improve the assessment of renal function. Conventional techniques produce clinical chemistry measures, but may generate an overestimate and fail to target the disease location. In contrast, DCE-MRI scanning enables the accurate classification and evaluation of localised renal function without the usage of ionising radiation. The computer-aided analysis of DCE-MRI could generate reliable biomarkers for clinical practice, for which an essential prerequisite involves segmenting the whole kidney and renal compartments, such as the cortex and medulla. This paper presents a fully modular and automated framework with a translational impact for kidney parenchyma segmentation, incorporating 3D deep learning and contrast enhancement of renal contextual information in the temporal dimension. Unlike the previous literature in renal compartment segmentation, the methodology proposed in this paper utilises a more extensive paediatric dataset and achieves outperforming quantitative accuracy scores, demonstrating stability in performance.

Considering limitations relating to a higher instance of higher false-negative or false-positive cortex segmentation in a subset of cases, future work will expand upon nature-inspired algorithms [39] to determine improved thresholding parameters. Another direction of future work will explore the usage of unsupervised deep learning for renal compartment segmentation, especially in light of limited ground-truth data. Methods that include feature hierarchy [40], deep representation [41] and autoencoders [42] will be investigated. Moreover, given the high level of motion-related artefacts, the advantage of developing a noise removal or suppression technique using deep learning [43] could have a significant impact on the resultant segmentation accuracy.

## Figures and Tables

**Table 1:** Comparison of the whole kidney segmentation accuracies.

Kidney Condition	Accuracy Result	Proposed Approach	3D Rb-UNet + 3D U-Net [26]
All	DSC	88.20 ± 6.41	84.41 ± 7.87
	PC	87.24 ± 6.37	83.24 ± 7.25
	RC	89.46 ± 7.90	86.49 ± 10.4
Normal	DSC	89.77 ± 4.79	85.30 ± 8.49
	PC	87.69 ± 6.15	83.98 ± 7.02
	RC	92.20 ± 5.16	87.98 ± 12.0
Abnormal	DSC	85.70 ± 7.75	82.97 ± 8.95
	PC	86.52 ± 6.64	82.05 ± 7.45
	RC	85.07 ± 9.41	84.09 ± 6.56

Quantitative accuracies obtained using the proposed approach and the state-of-the-art 3D U-Net [26] approach in terms of the mean dice similarity coefficient (DSC), precision (PC) and recall (RC) ± standard deviation (SD).

**Table 2:** Individual medulla and cortex segmentation accuracies.

Kidney Condition	DCE-MRI Case	Compartment	Proposed	Yoruk et al. [16]
Normal	1	Medulla	73.67	66.83
		Cortex	88.28	79.61
	2	Medulla	64.62	23.86
		Cortex	83.01	26.70
	3	Medulla	63.56	1.665
		Cortex	78.50	21.43
	4	Medulla	60.08	63.25
		Cortex	75.90	81.79
	5	Medulla	67.54	69.03
		Cortex	86.10	71.75
	6	Medulla	63.36	36.18
		Cortex	66.63	29.67
	7	Medulla	69.90	65.20
		Cortex	92.32	72.07
	8	Medulla	54.08	47.20
		Cortex	83.25	75.31
	9	Medulla	68.02	0.831
		Cortex	86.84	17.95
	10	Medulla	68.56	74.97
		Cortex	88.41	78.18
	11	Medulla	67.60	2.933
		Cortex	89.59	38.65
	12	Medulla	54.17	48.95
		Cortex	61.62	53.62
	13	Medulla	51.04	65.67
		Cortex	76.64	79.52
	14	Medulla	63.51	65.49
		Cortex	86.64	72.92
	15	Medulla	53.43	61.57
		Cortex	87.50	78.19
	16	Medulla	55.29	63.57
		Cortex	78.68	63.06
Abnormal	1	Medulla	67.34	64.52
		Cortex	74.54	74.57
	2	Medulla	63.16	61.06
		Cortex	82.88	76.21
	3	Medulla	64.40	63.90
		Cortex	89.08	65.94
	4	Medulla	69.44	72.76
		Cortex	89.00	72.17
	5	Medulla	61.17	72.44
		Cortex	84.29	83.62
	6	Medulla	62.40	49.79
		Cortex	80.98	59.27
	7	Medulla	69.37	69.51
		Cortex	87.13	78.21
	8	Medulla	59.56	59.29
		Cortex	74.14	76.28
	9	Medulla	51.18	50.55
		Cortex	70.64	79.41
	10	Medulla	66.11	69.54
		Cortex	86.37	79.30

A total of 16 clinically “normal” and 10 clinically “abnormal” cases (4D DCE-MRI volumes) were evaluated, and the individual dice similarity coefficient (DSC) results are listed.

**Table 3:** Mean medulla and cortex segmentation accuracies.

	Compartment	Proposed (%)	Yoruk et al. [16] (%)
**N-** **16 cases**	Cortex	81.87±8.21	58.78±22.85
	Medulla	62.40±6.69	47.32±25.27
**A-** **10 cases**	Cortex	81.90±6.31	74.50±6.79
	Medulla	63.41±5.16	63.34±7.86

Comparison of the proposed approach and a baseline approach referred to as Yoruk et al. [16]. A total of 16 clinically “normal” (N) and 10 clinically “abnormal” (A) 4D DCE-MRI volumes were evaluated. The results are presented as the mean dice similarity coefficient (DSC) ± standard deviation (SD).

**Figure A1:**
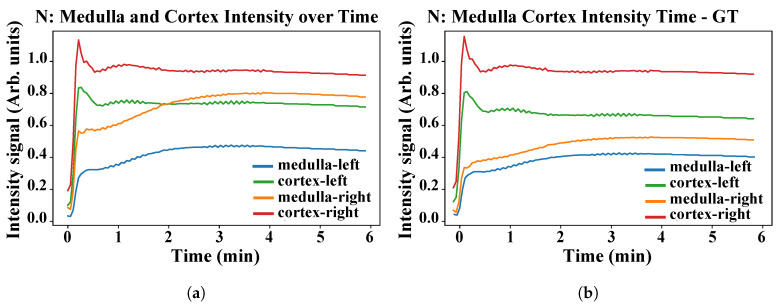

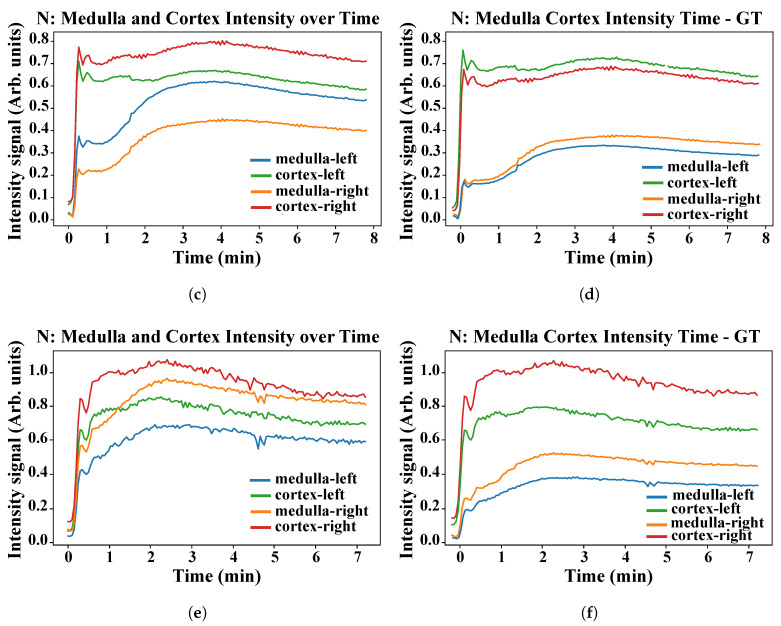
Graphs (**a**–**f**) represent the time–intensity curves of three clinically “normal” cases (scans or 4D volumes). The first column shows the relative contrast intensity enhancement of the automatic renal compartment segmentation in both the left and right kidneys over time (minutes). The second column shows the corresponding ground-truth (GT) time–intensity curves.

**Figure A2:**
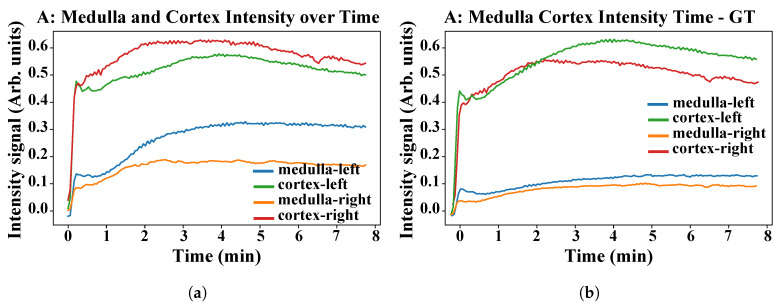

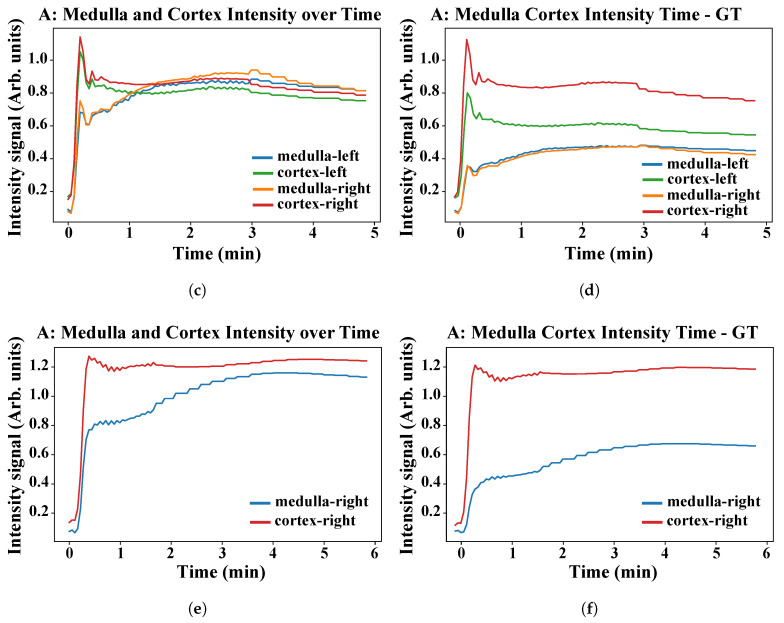
Graphs (**a**–**f**) represent the time–intensity curves of three clinically “abnormal” cases (scans or 4D volumes). The first column shows the relative contrast intensity enhancement of the automatic renal compartment segmentation in both the left and right kidneys over time (minutes). The second column shows the corresponding ground-truth (GT) time–intensity curves.

## Appendix A

As initially referenced in Section 4, Figure A1 and Figure A2 display the change in greyscale intensity over time using the automatic medulla and cortex compartment segmentation and ground truth for clinically “normal” and “abnormal” cases, respectively, indicating the rate at which the gadolinium contrast agent reaches the kidney.
